# The complete mitochondrial genome of the cavity-nesting honeybee, *Apis koschevnikovi* (Insecta: Hymenoptera: Apidae)

**DOI:** 10.1080/23802359.2016.1275847

**Published:** 2017-01-17

**Authors:** Takeshi Wakamiya, Salim Tingek, Hisashi Okuyama, Takuya Kiyoshi, Jun-ichi Takahashi

**Affiliations:** a Department of Life sciences, Kyoto Sangyo University, Kyoto, Japan;; b Agriculture Research Station Tenom, Tenom, Sabah, Malaysia;; c Department of Zoology, National Museum of Nature and Science, Tsukuba, Ibaraki, Japan

**Keywords:** Asian honeybee, Illumina sequencing, conservation, Borneo, *Apis koschevnikovi*

## Abstract

In this study, we analyzed the complete mitochondrial genome of the cavity-nesting honeybee, *A. koschevnikovi*. The mitochondrial genome of *A. koschevnikovi* was observed to be a circular molecule of 15,278 bp and was similar to that of the other cavity-nesting honeybee species. The average AT content in the *A. koschevnikovi* mitochondrial genome was 84%. It was predicted to contain 13 protein-coding, 24 tRNA and two rRNA genes, along with one A + T-rich control region, besides three *tRNA*-*Met* repeats.

The Southeast Asian honeybee, *Apis koschevnikovi*, belonging to subgenus *Apis* is a cavity-nesting honeybee of Asia belonging to a group that includes *A. cerana*, *A. nigrocincta*, and *A. nuluensis* along with the European-African *A. mellifera*. Molecular phylogenetic analyses of the partial DNA sequences indicate that this species and *A. cerana* are sister taxa (Arias et al. 1996; Tanaka et al. 2001). The distribution of this species has been geographically restricted, mainly in the isolated tropical evergreen straits of Borneo, Malay Peninsula and Sumatra (Hadisoesilo et al. 2008). Basic knowledge on this species has been lacking; little information is available on the genetic diversity and population density, which is necessary for conservation of the species (Tanaka et al. 2003).

Adult worker was collected from a hive in an apiary at the Agriculture Research Station Tenom in Sabah, Malaysia (the specimen is stored in the National Museum of Nature and Science, Japan accession number: NSMT-I-HYM742391). Genomic DNA isolated from one worker was sequenced using Illumina’s Next Seq 500 (Illumina). The resultant reads were assembled and annotated using the MITOS web server (Bernt et al. 2013, Germany), the MEGA6 software (Tamura et al. 2013) and GNETYX v.10 (Genetyx Corporation, Japan). The phylogenetic analysis was performed using the TREEFINDER v.2011 (Jobb et al. 2004) based on the nucleotide sequences of 13 protein-coding genes.

The *A. koschevnikovi* mitochondrial genome forms a closed loop that is 15,278 bp long (accession number AP017643). The *A. koschevnikovi* mitochondrial genome represents a typical hymenopteran mitochondrial genome and matches the common organization present in the bees; it comprises of 13 protein-coding, 24 putative tRNA, and two rRNA genes, along with an A + T-rich control region. However, the number of *tRNA*-*Met* (three) in *A. koschevnikovi* was two more than the number present in other cavity-nesting honeybees. Similar to other honeybee mitochondrial genomes, the heavy strand (H-strand) was predicted to contain nine protein-coding genes and 16 tRNA genes. The light strand (L-strand) was predicted to contain four protein-coding, eight tRNA, and two rRNA genes. All the tRNA genes possessed cloverleaf secondary structures. The *ATP6* and *ATP8* genes shared 19 nucleotides. Eight protein-coding genes of the *A. koschevnikovi* mitochondrial genome started with ATT, and *ATP6*, *COIII, ND4* and *Cytb* genes started with ATG. The stop codon of each of these protein-coding genes was either TAA or TAG, as observed in the case of other honeybees.

Phylogenetic analysis was performed using the sequences of 13 mitochondrial protein-coding genes and those of 14 closely related taxa (Figure 1). The phylogenetic analyses of the complete mitochondrial DNA genes strongly supported the result obtained from the phylogenetic analysis of partial DNA sequences (Arias & Sheppard 2005), grouping the monophyletic species within the cavity-nesting honeybees: *A. mellifera* (Crozier & Crozier 1993; Haddad 2015; Gibson & Hunt 2016; Hu et al. 2016), *A. koschevnikovi,* and *A. cerana* (Tan et al. 2011; Takahashi et al. 2016). The complete sequence of the *A. koschevnikovi* mitochondrial genome provides additional genetic tools for studying conservational genetics and biogeography of this species.

**Figure 1.**
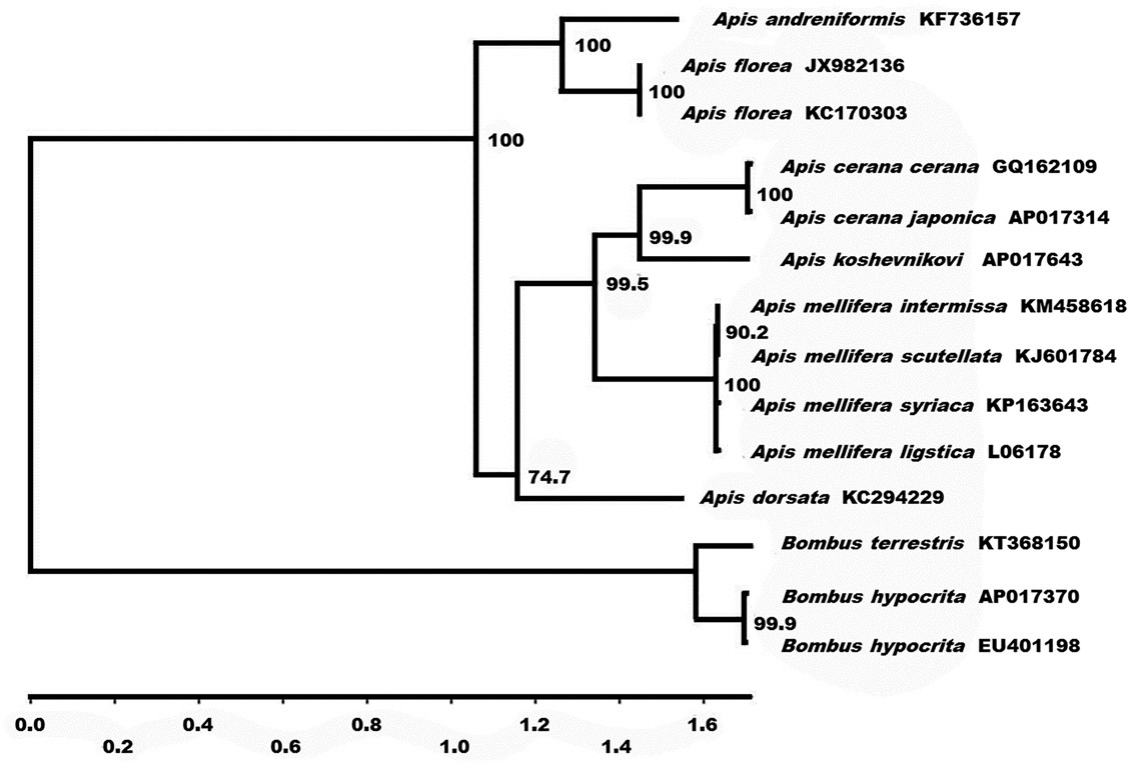
Phylogenetic relationships (maximum likelihood) of the genus *Apis* and *Bombus* of among the members of Apinae based on the nucleotide sequence of 13 protein-coding genes regions in the mitochondrial genome. The numbers beside the nodes are percentages of 1000 bootstrap values. The *Bombus terrestris* and *B. hypocrita* were used as an outgroup. Alphanumeric terms indicate the GenBank accession numbers.
